# Smartphone-based ecological momentary assessment reveals an incremental association between natural diversity and mental wellbeing

**DOI:** 10.1038/s41598-024-55940-7

**Published:** 2024-04-16

**Authors:** Ryan Hammoud, Stefania Tognin, Michael Smythe, Johanna Gibbons, Neil Davidson, Ioannis Bakolis, Andrea Mechelli

**Affiliations:** 1 Department of Psychosis Studies, Institute of Psychiatry, Psychology and Neuroscience, King’s College London, De Crespigny Park, London, SE5 8AF UK; 2 Nomad Projects, Sunbury Workshops, 24, Swanfield St, London, E2 7LF UK; 3 J & L Gibbons, 19 Swan Yard, London, N1 1SD UK; 4 Health Services and Population Research Department, Centre for Implementation Science, Institute of Psychiatry, Psychology and Neuroscience, King’s College London, London, UK; 5 Department of Biostatistics and Health Informatics, Institute of Psychiatry, Psychology and Neuroscience, King’s College London, London, UK

**Keywords:** Biodiversity, Nature, Mental wellbeing, Smartphones, Ecological momentary assessment, Urban environment, Environmental social sciences, Environmental impact, Psychology and behaviour, Psychology

## Abstract

Using smartphone-based ecological momentary assessment, this study investigated an association between natural diversity on mental wellbeing. A sample of 1,998 participants completed 41,448 assessments between April 2018 and September 2023. Environments which included a larger range of natural features, such as trees, plants and birdlife (high natural diversity) were associated with greater mental wellbeing than environments including a smaller range of natural features (low natural diversity). There was evidence of a mediating effect of natural diversity on the association between natural environments and mental wellbeing. These results highlight the importance of policies and practices that support richness of biodiversity for public mental health.

## Introduction

Recent reports suggest that up to 70% of the global population is expected to live in densely populated cities by the year 2050^1^. While urban living may benefit people’s wellbeing through improved access to social networks, health infrastructure, health policies and employment opportunities^2–6^, people residing in cities also experience an increased risk and incidence of mental illness^5,7–10^. The association between increased incidence of mental illness and city living has been linked to reduced exposure to nature^11–13^; conversely, there is a growing body of empirical evidence indicating that increased levels urban nature is associated with positive mental health outcomes^2,12^.

Two predominant theories have been proposed to explain these associations, each focusing on different aspects of how nature impacts mental health. Attention Restoration Theory suggests that exposure to nature restores cognitive functioning often diminished due to the demands of urban living^14,15^. In contrast, Stress Recovery Theory proposes that exposure to natural environments can alleviate physiological stress, serving as a buffer against urban stressors^16–18^. While the vast majority of research has investigated urban nature as a whole, some studies have reported individual associations between specific natural features within the urban environment, such as trees, gardens, parks, birdlife, and waterways, and increased mental wellbeing and reduced incidence of mental illness^19–23^. This pattern of results provides a rationale for the development of nature-based interventions to support the mental health of urban populations. However, the development of such interventions requires us to understand how individual natural features interact with each other to influence mental health. One possibility is that different natural features affect mental wellbeing in an incremental manner; in this case, environments which include a larger range of natural features, such as trees, plants, waterways, and wildlife (high natural diversity), would bring greater mental health benefits than environments which include a smaller range of natural features (low natural diversity). An alternative possibility is that natural features are associated with higher mental health irrespective of the amount of natural diversity; in this case, environments which include a larger range of natural features would have similar mental health benefits to environments which include a smaller range of natural features.

With regards to these two possibilities, the existing literature is surprisingly sparse. While several studies have investigated the concept of a minimum dose of nature^15,24–27^, a majority of them have primarily focused on visit frequency and duration, as well as the amount or proximity to green space. This approach tends to overlook natural diversity, a biological characteristic commonly assessed in urban ecology^28^. For instance, urban parks and golf courses offer access to green space but may not necessarily promote natural diversity.

A few studies investigating the effects of natural diversity have found positive associations with health and wellbeing through several distinct mechanistic pathways^29–32^. Increased natural diversity has supported human wellbeing by encouraging health-promoting behaviors like physical activity and social interaction^31^, protecting against chronic inflammatory disorders such as allergies and asthma^33–36^, reducing air pollution^37^, as well as through provision of ecosystem services like temperature regulation in urban areas or increased food production^38–40^. However, some studies found either no evidence or evidence of inverse relationships^30,31,41^. Huynen and colleagues^42^ found a positive association between percentage of threatened species and life expectancy and disability adjusted life expectancy. Hough^30^ suggests that, on a global scale, the correlation between these factors could be attributed the simultaneous rise in economic and industrial development, which poses a threat to species diversity, while also leading to an increase in human life expectancy.

Less understood are the impacts of biodiversity on mental wellbeing. Very few studies investigated the effect of a minimum dose in terms of natural diversity on mental wellbeing, and the specific characteristics of greenspaces that offer the greatest benefit^28,29,32,39^. A handful of studies have found psychological wellbeing increased with measured species richness and tree/plant coverage of green spaces^24,43–45^. On the other hand, Dallimer and colleagues^28^ found an association between mental wellbeing and perceived richness plant and animal species but not with actual richness. The same authors found no association between perceived diversity and objective diversity. This suggests limited biodiversity identification skills amongst people who live in developed countries where wildlife is scarce^28,46^ and highlights the importance of further research on the impact of natural diversity and mental wellbeing. Additionally, recent studies utilizing virtual environments of differing levels of natural diversity have shown non-linear effects on wellbeing, with some suggesting that the most substantial improvements on mental health outcomes were not always associated with the highest levels of biodiversity^47–49^.

The aim of the present study was to investigate the impact of natural diversity on self-reported mental wellbeing, a strong predictor of mental health in the general population. Here we define natural diversity based on the perceived number of different natural features (e.g. trees, plants, birds, water) within the surrounding environment. This approach allows us to examine whether a combination of different natural elements, rather than just the presence of green space or actual species richness, has an increased effect on mental wellbeing. This may be particularly relevant given the current limited understanding of the impacts of natural diversity on mental wellbeing.

While the bulk of existing research on the impact of nature on mental health has mainly focused on nature as a whole, many of these studies have been hindered by methodological limitations. Traditional approaches, such as surveys and questionnaires, depend heavily on participants recalling past experiences, inherently subject to recall bias^50^. Additionally, some studies have relied on artificial settings, presenting nature images to participants in controlled environments^51,52^, which may lack ecological validity and limit the applicability of findings to real-life contexts. Moreover, many of these studies employ cross-sectional designs which capture a single moment in time, failing to consider the dynamic nature of interactions with a variety of environments throughout the day^53^.

To overcome the limitations of the existing literature, the current study utilized smartphone-based ecological momentary assessment^54^. Firstly, this methodology allowed us to explore the impact of natural diversity on mental wellbeing in real-time, minimizing the risk of recall bias. Secondly, it enables repeated measurements over time, which allowed us to capture dynamic changes in mental wellbeing in a way that single-timepoint studies are unable to achieve. Thirdly, it facilitates the collection of rich collection of data on a variety of environments that individuals experience over time.

Using the Urban Mind smartphone application, we were able to gather comprehensive information on the type of natural features that individuals experience within their daily lives. This information was used to address the following questions:

1. Are momentary encounters with specific natural features, such as birdlife, trees, plants and water, associated with greater mental wellbeing? Are these associations time-lasting?
2. Is there an *incremental* association between the number of different natural features and mental wellbeing, suggesting greater mental health benefits in environments with higher natural diversity compared to those with lower natural diversity? Does this association endure over time?
3. How much of the association between momentary encounters with nature and mental wellbeing is mediated by the number of different natural features within the surrounding environment?

We hypothesized that being able to see or hear birdlife, trees, plants, and water would be associated with higher momentary mental wellbeing and that this effect would still be evident after the encounter had taken place (hypothesis 1). Secondly, we hypothesize that increasing the number of different natural features that individuals can see or hear would be positively associated with momentary mental wellbeing, and this association would be time-lasting (hypothesis 2). Lastly, we hypothesize that the number of different natural features that individuals can see or hear would have a significant mediating effect on the association between being in natural environments and momentary mental wellbeing (hypothesis 3).

## Methods

The current study received institutional review board (IRB) approval from the Psychiatry, Nursing and Midwifery Research Ethics Subcommittee at King’s College London (LRS-17/18–6905). All research was performed in accordance with relevant guidelines and regulations.

### Design

An observational study using data collected from the Urban Mind smartphone application^19^, a smartphone-based ecological momentary assessment (EMA) tool available for both Apple iPhone and Android devices.

### The Urban Mind app

Urban Mind is a smartphone app used for measuring the impact of the built and social environment on mental wellbeing. It was developed as part of the Urban Mind research project—a collaboration between King’s College London, landscape architects J&L Gibbons and arts foundation Nomad Projects. Extensive information about an earlier version of the Urban Mind app can be found elsewhere^19^, but a brief summary of the version adapted for the current study is provided here. Participants were recruited globally over a period of 65 months (April 2018–September 2023) using various social media platforms, the project-related website, and word of mouth. Participation in the study was self-selected and anonymous. After downloading and installing the app, individuals were presented with details about the study and were asked to provide informed consent. Once informed consent was obtained, participants were then asked to complete an initial assessment. This baseline assessment gathered data on demographics (e.g. age, gender and ethnicity), socioeconomic factors (e.g. education, occupation), sleep patterns (e.g. typical waking and sleeping times), and self-reported mental health history (e.g. current and past mental health diagnoses). Following the initial baseline assessment, the app scheduled a total of 42 ecological momentary assessments over the subsequent 14 days, with three assessments per day. These assessments were scheduled to align with each participant’s self-reported sleep schedule. The time when participants were awake was divided into three equal intervals, and a random assessment was scheduled within each time window. Once an assessment became available, the app would notify the participant to respond within one hour before marking it as incomplete. This approach allowed data to be captured from individuals at different times of the day and at a variety of locations and contexts, while minimizing the burden of repeated assessments and disruption to their daily routines. These momentary assessments collected information about participants’ perceptions of their subjective built and social environment, and their location via GPS-based geotagging. At the end of each assessment, participants were prompted to capture and submit a photograph and 8-s audio clip of their environment. While these images and audio clips were not included in the statistical analysis, they served to increase participant engagement and were shared on our social media platforms and website (www.urbanmind.info).

#### Participants

During the 65-month recruitment period (April 2018–September 2023), 7829 participants download the Urban Mind app and completed the baseline assessment. Of this sample, 1998 participants completed at least 25% of the assessments (a minimum of 11 out of 42 assessments), 922 participants completed at least 50% of the assessments (a minimum of 21 out of the 42 assessments), and 310 participants completed at least 75% of the assessments (32 out of the 42 assessments) (Table 1).

**Table 1:** Sociodemographic characteristics of the main and sensitivity samples.

Assessment response rate	≥ 11 out of 42 assessments (≥ 25%)	≥ 21 out of 42 assessments (≥ 50%)	≥ 32 out of 42 assessments (≥ 75%)
	Number (%)	Number (%)	Number (%)
Number of participants	*n* = 1,998	*n* = 922	*n* = 310
Gender			
Female	1413 (70.9%)	651 (70.8%)	208 (67.3%)
Male	557 (28.0%)	262 (28.5%)	99 (32.0%)
Other	23 (1.2%)	7 (0.8%)	2 (0.7%)
Age	Mean: 36.5.5 SD: 14.5 Range: 16–82	Mean: 36.9 SD: 14.8 Range: 16–77	Mean: 37.7 SD: 15.6 Range: 16–73
Ethnicity			
White/Caucasian	1353 (68.2%)	622 (67.7%)	205 (66.6%)
Asian	356 (17.9%)	170 (18.5%)	69 (22.4%)
Other	276 (13.9%)	127 (13.8%)	34 (11.0%)
Occupation			
Student	525 (26.3%)	255 (27.7%)	89 (28.7%)
Employed	1071 (53.6%)	494 (53.6%)	157 (50.7%)
Self-employed	212 (10.6%)	79 (8.6%)	28 (9.0%)
Retired	107 (5.4%)	54 (5.9%)	26 (8.4%)
Unemployed	83 (4.2%)	40 (4.3%)	10 (3.2%)
Education			
Less than high school	32 (1.6%)	15 (1.6%)	2 (0.7%)
High school	215 (10.8%)	99 (10.7%)	37 (11.9%)
Apprenticeship	143 (7.2%)	72 (7.8%)	24 (7.7%)
University	1608 (80.5%)	736 (79.8%)	247 (79.7%)
	Observations (%)	Observations (%)	Observations (%)
Number of Momentary Assessments	*n* = 41,488	*n* = 26,049	*n* = 10,722
Can you see trees?			
No	17,119 (41.3%)	10,563 (40.5%)	4,328 (40.4%)
Yes	24,369 (58.7%)	15,486 (59.5%)	6,394 (59.6%)
Can you see plants?			
No	15,205 (36.7%)	9,472 (36.4%)	3,858 (36.0%)
Yes	26,269 (63.3%)	16,573 (63.6%)	6,859 (64.0%)
Can you see or hear birds?			
No	29,588 (71.8%)	18,498 (71.5%)	7,438 (70.0%)
Yes	11,629 (28.2%)	7,387 (28.5%)	3,194 (30.0%)
Can you see water?			
No	36,533 (88.2%)	22,913 (88.1%)	9,243 (86.4%)
Yes	4,879 (11.8%)	3,095 (11.9%)	1,451 (13.6%)
Momentary mental wellbeing	Mean: 34.8 SD: 7.16	Mean: 35.1 SD: 7.09	Mean: 35.7 SD: 7.05
Natural diversity score	Mean: 1.62 SD: 1.23	Mean: 1.63 SD: 1.24	Mean: 1.67 SD: 1.26

*Number (%)* refers to the number and percentage of participants in each sample. *Observations (%)* refers to the number and percentage of completed ecological momentary assessment observations in each sample. The total of % and *n* may not reflect the full sample due to missing data or ‘Not sure’, or ‘Prefer not to say’ responses, excluded from the analyses.

The main sample, highlighted in grey, comprised of participants who completed at least 50% of momentary assessments and two sensitivity samples comprised of those who completed at least 25% and 75% momentary assessments, respectively.

### Measures

#### Mental wellbeing

Momentary mental wellbeing scores were assessed as the sum of five positive affect items and the reverse of five negative affect items. The positive statements included: 1) Right now I am feeling confident; 2) Right now I am feeling relaxed; 3) Right now I am feeling happy; 4) Right now I am feeling connected with other people; 5) Right now I am feeling energetic; and the negative statements included: 1) Right now I am feeling anxious; 2) Right now I am feeling stressed; 3) Right now I am feeling down; 4) Right now I am feeling lonely; 5) Right now I feeling tired. Participants were asked to indicate their level of agreement to the statements on a 5-point Likert scale ranging from “Strongly disagree” (1) to “Strongly agree” (5).

#### Natural diversity score

A natural diversity score was derived from the sum of the four natural features explored during each Urban Mind assessment—(1) Can you see trees right now? (2) Can you see plants right now? (3) Can you see or hear birds right now? (4) Can you see or hear water right now? A “No” response was given a value of 0 and a “Yes” response was given a value of 1. Natural diversity score ranged from 0 (no natural diversity) to 4 (high natural diversity).

#### Natural environments

Exposure to natural spaces was a variable derived from the question “Where are you exactly?”. If a participant indicated they were at “Home”, “Public Place e.g. shop/restaurant/cinema, “Street/square”, “Public transport”, “School/University”, “Workplace”, or “Other” were rated as “No”. If a participant indicated they were “By sea/lake/river” or “Garden/park”, they were rated as “Yes”.

#### Confounders

Demographic characteristics such as age, gender, ethnicity, education, and occupational status were included as potential confounding variables. These were self-reported during the initial momentary assessments.

#### Statistical analysis

All statistical analyses were performed with STATA/MP 16. Longitudinal associations between self-reported contact with natural features, natural diversity and mental wellbeing were investigated using random intercept multilevel regression models and expressed as mean differences (MD) and 95% confidence intervals (CI). All models were adjusted for the following confounders: age, gender, ethnicity, education, and occupational status. Furthermore, time-lagged random intercept regression models were used to test for time-lasting associations between self-reported contact with natural features and diversity and mental wellbeing. In order to address missing data due to skipped assessments, all models were rerun using the STATA ice routine, an implementation of the Multiple Imputations with Chained Equations (MICE) procedure^55^. The results using the MICE procedure were then compared with the results from the original analysis under the missing at random (MAR) assumption^56^.

A parametric multilevel linear regression mediation approach (*ml_mediation* package in STATA/MP 16.0) was used to estimate the total effect, the natural indirect effects (NIE), and the natural direct effects (NDE) of being in natural environments on mental wellbeing (Table 4). The NDE represented the effect of being in natural environments on mental wellbeing that was independent of amount of natural diversity. An NIE represented the proportion of the effect of *being in natural environments on mental wellbeing* that could be explained by the *amount of natural diversity.* To ensure the robustness of these estimates while considering the total number of observations, a bootstrap method with 50 replications was employed.

## Results

### Are encounters with natural features of the environment associated with mental wellbeing?

Multilevel regression analyses showed significant positive associations between seeing or hearing birds seeing trees, seeing plants, seeing or hearing water and mental wellbeing (Table 2). These positive associations were found within our main and sensitivity 25% and 75% response rates. The associations remained significant after adjusting for potential confounders (age, gender, ethnicity, education, and occupation) and after implementing the MICE procedure to address missing data (Supplementary Table 1).

**Table 2:** Momentary associations between natural features and mental wellbeing.

	25% response rate (*n* = 1,998)		50% response rate (*n* = 922)		75% response rate (*n* = 310)	
	Unadjusted	Adjusted	Unadjusted	Adjusted	Unadjusted	Adjusted
	MD (95% CI)	MD (95% CI)	MD (95% CI)	MD (95% CI)	MD (95% CI)	MD (95% CI)
Seeing or hearing birds	1.43*** **(1.31, 1.54)**	1.41*** **(1.29, 1.53)**	1.59*** **(1.44, 1.74)**	1.58*** **(1.43, 1.73)**	1.57*** **(1.34, 1.81)**	1.56*** **(1.32, 1.79)**
Seeing trees	1.05*** **(0.94, 1.16)**	1.04*** **(0.93, 1.15)**	1.13*** **(1.00, 1.27)**	1.12*** **(0.99, 1.26)**	1.09*** **(0.87, 1.31)**	1.06*** **(0.84, 1.28)**
Seeing plants	1.28*** **(1.17, 1.39)**	1.27*** **(1.15, 1.38)**	1.32*** **(1.18, 1.46)**	1.32*** **(1.17, 1.46)**	1.31*** **(1.08, 1.54)**	1.31*** **(1.08, 1.54)**
Seeing or hearing water	1.47*** **(1.30, 1.63)**	1.45*** **(1.28, 1.62)**	1.64*** **(1.43, 1.85)**	1.63*** **(1.42, 1.84)**	1.62*** **(1.30, 1.93)**	1.60*** **(1.28, 1.92)**

Mean difference (MD) and 95% confidence intervals (CI) represent the mean difference in momentary mental wellbeing per category increase compared to the reference group.

Statistically significant associations (*p* < 0.05) are highlighted in bold.

Analyses were explored as crude associations and after adjusting for age, gender, ethnicity, education, and occupation.

*p < 0.05; **p < 0.01; ***p < 0.001.

### Are the associations between natural features of the environment and mental wellbeing time-lasting?

**Figure 1:**
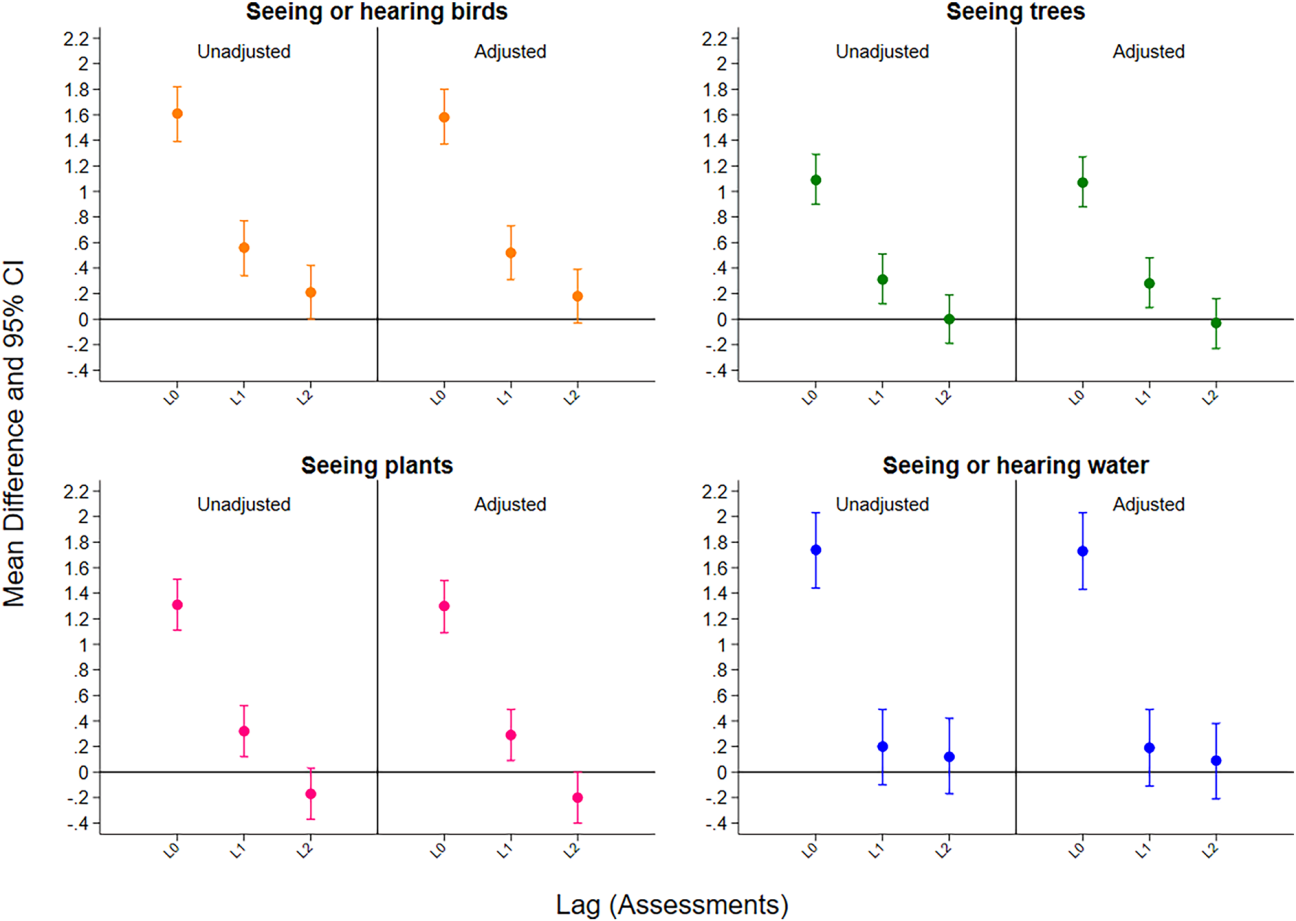
Time lasting associations between natural features and mental wellbeing in the 50% response rate main sample. Note: Mean difference (MD) and 95% confidence intervals (CI) represent the mean difference in momentary mental wellbeing per category increase compared to the reference group. L0–L2 represent the assessments lagged from 0 to 2. L0 indicates the impact of natural features on mental wellbeing at the time of the assessment; L1 indicates the impact of natural features on mental wellbeing at the during the first subsequent assessment (after an average of 8 h); L2 indicates the impact of natural features on mental wellbeing during the second subsequent assessment (after an average of 16 h). Analyses were explored as crude associations and after adjusting for age, gender, ethnicity, education, and occupation.

Time-lagged random intercept regression models demonstrated positive associations between seeing or hearing birds, seeing trees, and seeing plants during the subsequent assessment (Fig. 1; Supplementary Table 2). Seeing or hearing water did not have any significant time-lasting effects on mental wellbeing. These associations were replicated in the 25% response rate and 75% response rate samples, with the exemption of seeing plants at the 75% response rate. These associations were also replicated when adjusting for potential confounders, and after implementing the MICE procedures to address missing data (Supplementary Table 3).

### Is there an association between natural diversity and mental wellbeing? And is this time lasting?

Multilevel regression models showed significant positive associations between natural diversity score and mental wellbeing (Table 3). The mean differences (MD) in mental wellbeing scores were 0.74 (95%CI: 0.69, 0.80) within our main sample, 0.69 (95%CI: 0.65, 0.74) and 0.74 (95%CI: 0.65, 0.83) at the sensitivity 25% and 75% response rate samples. The associations remained significant after adjusting for potential confounders and after implementing the MICE procedure to address missing data (Supplementary Table 4).

**Table 3:** Associations between natural diversity score, exposure to natural environments, mental wellbeing, and their time-lasting effects.

	25% response rate (*n* = 1,998)		50% response rate (*n* = 922)		75% response rate (*n* = 310)	
	Unadjusted	Adjusted	Unadjusted	Adjusted	Unadjusted	Adjusted
	MD (95% CI)	MD (95% CI)	MD (95% CI)	MD (95% CI)	MD (95% CI)	MD (95% CI)
Total natural diversity score	0.69*** **(0.65, 0.74)**	0.68*** **(0.64, 0.73)**	0.74*** **(0.69, 0.80)**	0.74*** **(0.68, 0.80)**	0.74*** **(0.65, 0.83)**	0.73*** **(0.64, 0.82)**
Time-lasting effects						
L0	0.74*** **(0.67, 0.82)**	0.72*** **(0.65, 0.80)**	0.74*** **(0.66, 0.82)**	0.73*** **(0.65, 0.81)**	0.76*** **(0.65, 0.87)**	0.75*** **(0.64, 0.86)**
L1	0.21*** **(0.13, 0.28)**	0.18*** **(0.11, 0.25)**	0.20*** **(0.12, 0.28)**	0.18*** **(0.10, 0.26)**	0.23*** **(0.13, 0.34)**	0.22*** **(0.11, 0.33)**
L2	0.06 (− 0.01, 0.13)	0.04 (− 0.03, 0.11)	0.01 (− 0.07, 0.09)	0.00 (− 0.08, 0.08)	0.03 (− 0.08, 0.14)	0.01 (− 0.10, 0.12)
Natural environments	3.59*** **(3.32, 3.86)**	3.59*** **(3.32, 3.86)**	3.90*** **(3.56, 4.24)**	3.90*** **(3.56, 4.25)**	4.09*** **(3.58, 4.59)**	4.09*** **(3.58, 4.60)**
Time-lasting effects						
L0	3.58*** **(3.14, 4.02)**	3.58*** **(3.13, 4.02)**	3.69*** **(3.21, 4.17)**	3.70*** **(3.22, 4.18)**	4.15*** **(3.53, 4.78)**	4.18*** **(3.55, 4.81)**
L1	1.02*** **(0.59, 1.45)**	1.04*** **(0.61, 1.47)**	1.20*** **(0.72, 1.67)**	1.21*** **(0.74, 1.69)**	1.02** **(0.41, 1.63)**	1.04** **(0.43, 1.66)**
L2	0.35 (− 0.08, 0.78)	0.37 (− 0.06, 0.80)	0.32 (− 0.15, 0.80)	0.32 (− 0.15, 0.80)	0.51 (− 0.10, 1.13)	0.52 (− 0.10, 1.14)

Mean difference (MD) and 95% confidence intervals (CI) represent the mean difference in momentary mental wellbeing per category increase compared to the reference group.

L0–L2 represent the assessments lagged from 0 to 2. L0 indicates the impact of natural diversity or natural environments on mental wellbeing at the time of the assessment; L1 indicates the impact of natural diversity or natural environments on mental wellbeing at the during the first subsequent assessment (after an average of 8 h); L2 indicates the impact of natural diversity or natural environments on mental wellbeing during the second subsequent assessment (after an average of 16 h).

Statistically significant associations (*p* < 0.05) are highlighted in bold.

Analyses were explored as crude associations and after adjusting for age, gender, ethnicity, education, and occupation.

*p < 0.05; **p < 0.01; ***p < 0.001.

Using time-lagged random intercept regression models, we found a positive association between natural diversity score and momentary mental wellbeing during the subsequent assessment (_L1_MD: 0.20; 95%CI: 0.12, 0.28). While the positive association remained during the subsequent assessment, the effect was lower than at the time of exposure. The time-lasting effect was not evident during the second subsequent assessment. These results remained consistent in our sensitivity samples, after adjusting for potential confounders, and after implementing the MICE procedures to adjust for missing data (Supplementary Table 4).

### Is the association between being in natural environments and mental wellbeing mediated by amount of natural diversity?

**Figure 2:**
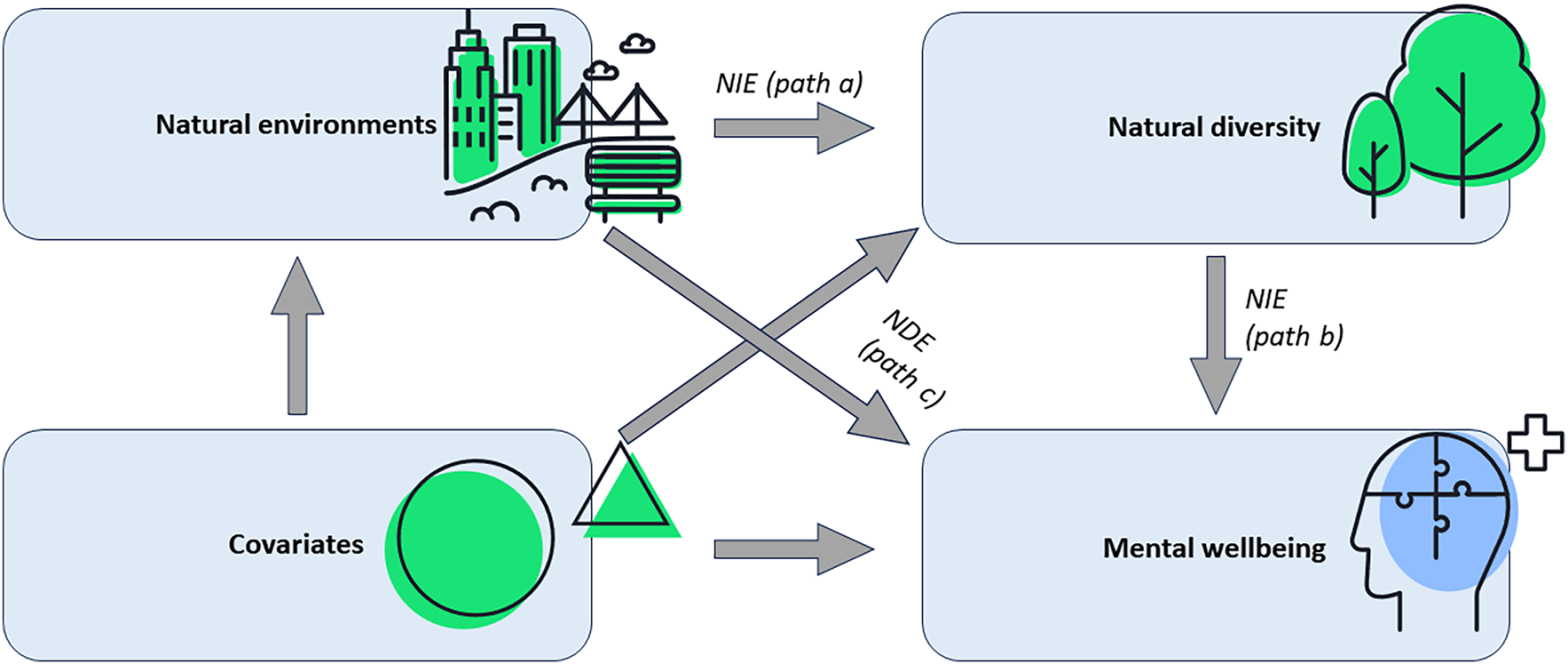
Direct acyclic graph of a structural model of mediation of the effect of natural environments on mental wellbeing by amount of natural diversity. *NDE* natural direct effects, *NIE* natural indirect effects.

The indirect effects via *amount of natural diversity* suggested a 0.91 (95%CI: 0.83, 0.99) increase in mental wellbeing when participants were *in natural environments* (Fig. 2; Table 4). The proportion of the association between *being in natural environments* and *mental wellbeing* mediated by *amount of natural diversity* was 23.4%. The direct effect of *being in natural environments* indicated that we would, on average, observe a 2.98 (95%CI: 2.61, 3.35) increase in total mental wellbeing if participants experienced low levels of natural diversity (Table 4).

**Table 4:** Adjusted direct and indirect effects of natural environments on mental wellbeing mediated by number of natural features.

Measure	25% response rate Mental wellbeing^¶^ (n = 1998)	50% response rate Mental wellbeing^¶^ (n = 922)	75% response rate Mental wellbeing^¶^ (n = 310)
	MD (95% CI)	MD (95% CI)	MD (95% CI)
Natural environments			
Total effect	3.58* (3.30, 3.85)	3.89* (3.51, 4.26)	4.06* (3.62, 4.51)
Direct effect	2.73* (2.45, 2.99)	2.98* (2.61, 3.35)	3.20* (2.77, 3.63)
Indirect effect via *amount of natural diversity*	0.86* (0.79, 0.92)	0.91* (0.83, 0.99)	0.86* (0.71, 1.01)
Proportion mediated (%)	23.9%	23.4%	21.2%
Total proportion mediated (%)	23.9%	23.4%	21.2%

Statistically significant associations (*p* < 0.05) are highlighted in bold.

*p < 0.05; **p < 0.001.

^a^Adjusted for baseline outcome/mediator and age, gender, ethnicity, education, and occupation.

^b^95% percentile intervals from bootstrapping with 50 replications.

^¶^Multi-level by individual (random intercept) mixed-effects linear regression.

## Discussion

Within existing literature, very few studies have examined the impact of natural diversity on mental wellbeing. To our knowledge, this is the first study to examine the impact of natural diversity on mental wellbeing as people go about their daily lives. This was achieved utilizing smartphone-based EMA, enabling us to repeatedly sample people’s experiences in real-time and in real-world contexts.

Consistent with our first hypothesis, we found that encounters with birdlife, seeing trees and plants, and seeing or hearing water all had a positive impact on mental wellbeing. These effects remained consistent across all three completion rate thresholds, as well as after adjusting for potential confounders including age, gender, ethnicity, education and occupation. Additionally, we found that the beneficial effects of encounters with birdlife, seeing trees and plants on mental wellbeing persisted even after the initial encounter. In contrast, we did not find any significant time-lasting effects of seeing or hearing water on mental wellbeing. This aligns with our earlier research on benefits of birdlife, green spaces, and blue spaces^19–21^ which demonstrated time-lasting benefits for mental health. These findings suggest that individual environmental features have both immediate and time-lasting distinct effects on mental wellbeing.

Our second hypothesis was that there would be an incremental association between natural diversity, the number of different natural features in the immediate environment, and mental wellbeing. This hypothesis was also confirmed, with natural diversity linked with an average 0.74 (95% CI: 0.68, 0.80) increased mental wellbeing score. In addition, the fact that we utilized smartphone-based EMA allowed us to repeatedly sample participants over time across different environments with varying levels of natural diversity. This also enabled us to test for time-lasting effects of varying levels of natural diversity within participants. Consistent with our hypothesis, we found that the positive association between natural diversity and mental wellbeing was time-lasting, suggesting that higher levels of natural diversity showed increased beneficial effects, both immediate and sustained, on mental wellbeing. Once again, this finding remained consistent across all three completion rate thresholds, as well as after adjusting for potential sociodemographic confounders including age, gender ethnicity, education and occupation. These results are in line with previous studies which found that species richness of urban greenspaces were associated with mental wellbeing^24,28,43–45^. Our study extends this existing literature by demonstrating that natural diversity affects momentary mental wellbeing and continues to benefit individuals for up to 8 h while taking individual characteristics into account.

Consistent with our third hypothesis, there was evidence that the association between natural environments and mental wellbeing was mediated by the amount of natural diversity. While being in natural environments had a significant positive impact on mental wellbeing independent of natural diversity, amount of natural diversity did account for 23.4% of the overall relationship. Specifically, for every additional natural feature, there was a 0.91 (95% CI: 0.83, 0.99) average increase in mental wellbeing when individuals were in natural environments. This indirect effect through natural diversity suggests that not just the presence of natural environments, but also their diversity, plays a crucial role in the impact on mental wellbeing.

These findings hold significant implications for both environmental conservation and urban planning policies. On the conservation front, the current study highlights the importance of protecting and promoting natural diversity in our cities. In practice, this means moving away from heavily curated monocultural pockets and parks of mown grass, which are typically associated with low biodiversity, towards polycultural spaces which mimic the biodiversity of natural ecosystems. In the current study, we defined natural diversity in terms of broad categories such as trees, plants, birds and water; the identification of these categories within one’s surrounding environment was a simple task which did not require specific biodiversity identification skills. Nevertheless, it is essential to recognize the nuanced relationship between perceived and objective species richness. Prior research has shown that individuals in developed countries may have limited biodiversity identification skills, resulting in a disconnection between perceived and actual ecological richness of a given green space^46^. In light of this disconnection, it has been suggested that conservation strategies should consider not only increasing actual biodiversity, but also enhancing understanding of biodiversity amongst the general public through education^28,39^.

Building on the concept of nature dose, our findings highlight the importance of developing health interventions which consider not only the minimum amount of nature but also the minimum level of natural diversity. The success of these interventions will require two interrelated strategies. First, national and local policies must protect and increase opportunities for momentary experiences of natural diversity within urban environments; second, meaningful engagement must be fostered amongst local communities through outreach and education initiatives aimed at knowledge-sharing and stewardship-upskilling for understanding and protecting urban biodiversity ecosystems.

It is also important to recognize that in densely populated areas like cities, promoting biodiversity can sometimes lead to human-environmental conflicts, ranging from minor conveniences, such as the presence of flies or spiders, to more significant concerns such as increased risk of Lyme disease^41^, lack of an accessible path or reduced feelings of safety^39^. However, it should also be recognized that low biodiversity poses its own challenges to human health, including increased vulnerability to trans-species transmission of disease and infectious agents^57^. Land management, maintenance and education efforts around residential areas with high levels of natural biodiversity can help create balanced coexistence between humans and the natural environment. Striking this balance is crucial as we work towards developing urban environments that support human health through increased biodiversity, while ensuring the safety and comfort of those living in the cities.

### Strengths

The current study offers several advantages over traditional methods of previous research. The Urban Mind app utilized EMA, a methodology involving the collection of real-time and real-world data by repeatedly sampling individuals’ experiences^54^. By implementing EMA within the app, we minimized a number of limitations that have hindered much of the existing literature.

Firstly, while a significant portion of the literature has focused on the effects of contact with green spaces such as forests or parks, many studies fail to consider the distinct characteristics within these environments. By overlooking these individual features, our understanding of the specific elements of nature that may impact mental wellbeing remains incomplete. Using the Urban Mind app, we were able to collect detailed information about specific natural features in the immediate environments of our participants.

Secondly, a substantial amount of prior research relies on the use of questionnaires or surveys that require participants to recall past experiences, which introduces the potential for recall bias^58^. Other studies employed artificial setups involving the presentation of images or videos to participants in front of computer screens. While these approaches may yield valuable insights, they may lack ecological validity, making it challenging to generalize the findings to real-life contexts. The Urban Mind app allowed us to collect information about participants’ surrounding environment and mental wellbeing in real-time in real-world contexts.

Furthermore, many studies have adopted a cross-sectional design, capturing information about a single point in time. This approach fails to account for the fact that individuals experience a diverse range of environments throughout the day, each with varying degrees of biodiversity. The Urban Mind app allowed us to repeatedly sample individuals over a span of 14 days to comprehensively understand the dynamic and time-lasting relationship between nature and mental well-being.

Lastly, the impact of green spaces on mental health may vary depending on individual characteristics, such as age, gender and lifestyle. However, many studies do not take these potential mediating factors into account, limiting our understanding of the differential effects of exposure to nature on diverse populations. We were also able to gather detailed information about the participants, enabling us to take into account the potential confounding effects of age, gender, ethnicity, education and occupation.

### Limitations

Firstly, the sample in the current study was self-selected, potentially introducing selection bias. Participants who voluntarily downloaded and engaged with the Urban Mind app likely have a specific interest in the environment’s impact on wellbeing, potentially limiting the generalizability of our findings. Despite this, as the app was available across both Apple iPhone and Android platforms to the general population, this ensured participation from various countries, suggesting enhancing the global relevance. As participants were aware of the study’s aim to investigate the effects of the environment on mental wellbeing, this may have introduced bias within their responses due to increased self-awareness.

Another limitation relates to the demographic characteristics of our sample, entirely smartphone users with an average age of 35.5 years, with a majority possessing a university-level education and employment or student status. This demographic profile may not fully represent the general population, and thus caution must be taken when generalizing our findings to diverse demographic groups. Additionally, our study relied on self-report measures, a source for potential bias^59^, as individuals may be more likely to complete assessments when they are in better moods or more pleasant environments, and employed an observational design, making it challenging to establish direct causal relationships between natural environments and mental wellbeing. While we have identified significant associations, due to the observational nature of the study, they should not be interpreted as definitive proof of causation. Furthermore, the study also faces limitations related to the mental wellbeing score used. This score, designed specifically for the Urban Mind app, is based on five positive and five negative affect items, aiming for ease and speed of completion to minimize participant burden. While this approach is widely used in EMA studies, it is important to recognize that our score was not previously validated. Additionally, we acknowledge the exclusion of certain environmental and lifestyle factors such as urban versus rural contexts, weather conditions, noise levels, and physical activity, which may play important roles in the interaction between nature and mental wellbeing. Future research would benefit from incorporating these aspects, alongside validating the mental wellbeing scale, to provide a more comprehensive view of the influences on mental health in varied settings.

Lastly, biodiversity measurement in urban settings is complex, with various approaches, potentially leading to different interpretations of the impact on mental wellbeing. While some studies utilize species richness^28,43^, others use indicators of ecological performance such as vegetation cover and density^24,58^, types of green space^44^ and intra-species diversity^45^. In the current study we defined natural diversity as the number of distinct perceived natural features in one’s environment (e.g., trees, plants, birds, water). This simple measure of natural diversity is likely to be associated with more nuanced measures of biodiversity, such as intra-species richness. Nevertheless, while a handful of existing studies have found a positive link between wellbeing and objective species richness^24,43–45^, other research reported associations with subjective richness, but not with objective measures^28^. This highlights that the context of how individuals perceive and interact with natural diversity may significantly influence diversity and wellbeing. Future studies would benefit from using and comparing different measures of perceived and objective biodiversity. This could offer a better understanding of the mechanisms underlying the association with mental wellbeing and provide deeper insights into the impact of different aspects of biodiversity, including intra-species and inter-species diversity. Such advancements in understanding could play a pivotal role in evidence-based policies and interventions aimed at promoting public mental health.

## Conclusion

Our study highlights the importance of considering both the accessibility of natural environments and the richness of biodiversity within them when designing spaces aimed at improving mental health. To our knowledge, this is the first study examining the mental health impact of everyday encounters with varying levels of natural diversity in real-time and real-life contexts. We found significant evidence supporting the time-lasting positive impact of individual natural features on mental wellbeing. We also report a significant time-lasting incremental effect of natural diversity on mental wellbeing. Given the complex nature of biodiversity measurement in urban areas, there is a need for future studies to explore the comparison between varying objective measures and perceived environmental features. Furthermore, future research should also consider employing experimental designs, as well as developing targeted interventions to provide a deeper understanding and complement the evidence base around the benefits of natural diversity for mental wellbeing. Our study has data-driven implications for conservation, urban planning and wellbeing policy. It highlights the importance for urban planners, policy makers, and mental health professionals to design, maintain, and promote the use of diverse green spaces in urban areas that not only enhance ecosystem biodiversity, but also facilitate improved human-nature coexistence, ultimately enhancing the wellbeing of local communities.

### Supplementary Information

Supplementary Tables.

## Data Availability

The data generated and analyzed for the current study and are not publicly available due to further analyses being planned, but de-identified data may be made available from the corresponding author on reasonable request.

## Abbreviations

EMA: Ecological momentary assessment
MICE: Multiple imputations with chained equations
MAR: Missing at random
NIE: Natural indirect effects
NDE: Natural direct effects

## References

[1] World Health Organization. *Urban Health*. https://www.who.int/news-room/fact-sheets/detail/urban-health (2021).

[2] Gascon M (2015). Mental health benefits of long-term exposure to residential green and blue spaces: A systematic review. Int. J. Environ. Res. Public Health.

[3] Dye C (2008). Health and urban living. Science.

[4] Galea S, Vlahov D (2005). URBAN HEALTH: Evidence, challenges, and directions. Annu. Rev. Public Health.

[5] Peen J, Schoevers RA, Beekman AT, Dekker J (2010). The current status of urban–rural differences in psychiatric disorders. Acta Psychiatr. Scand..

[6] Rydin Y (2012). Shaping cities for health: Complexity and the planning of urban environments in the 21st century. Lancet.

[7] Lederbogen F, Haddad L, Meyer-Lindenberg A (2013). Urban social stress–risk factor for mental disorders. The case of schizophrenia. Env. Pollut.

[8] Vassos E, Pedersen CB, Murray RM, Collier DA, Lewis CM (2012). Meta-analysis of the association of urbanicity with schizophrenia. Schizophr. Bull..

[9] Haddad L (2015). Brain structure correlates of urban upbringing, an environmental risk factor for schizophrenia. Schizophr. Bull..

[10] Weich S (2002). Mental health and the built environment: Cross-sectional survey of individual and contextual risk factors for depression. Br. J. Psychiatry.

[11] Sarkar C, Gallacher J, Webster C (2013). Urban built environment configuration and psychological distress in older men: Results from the Caerphilly study. BMC Public Health.

[12] Bratman GN (2019). Nature and mental health: An ecosystem service perspective. Sci. Adv..

[13] Cox DTC, Hudson HL, Shanahan DF, Fuller RA, Gaston KJ (2017). The rarity of direct experiences of nature in an urban population. Landsc. Urban Plan..

[14] Kaplan, R. *The Nature of the View from Home: Psychological Benefits*. *Environment and Behavior* vol. 33 (2001).

[15] White MP (2021). Associations between green/blue spaces and mental health across 18 countries. Sci. Rep..

[16] Ulrich RS (1979). Visual landscapes and psychological well-being. Landsc. Res..

[17] Ulrich RS (1981). Natural versus urban scenes: Some psychophysiological effects. Environ. Behav..

[18] Ulrich RS (1991). Stress recovery during exposure to natural and urban environments. J. Environ. Psychol..

[19] Bakolis I (2018). Urban mind: Using smartphone technologies to investigate the impact of nature on mental well-being in real time. Bioscience.

[20] Bergou N (2022). The mental health benefits of visiting canals and rivers: An ecological momentary assessment study. PLoS ONE.

[21] Hammoud R (2022). Smartphone-based ecological momentary assessment reveals mental health benefits of birdlife. Sci. Rep..

[22] Völker S, Kistemann T (2015). Developing the urban blue: Comparative health responses to blue and green urban open spaces in Germany. Heal. Place.

[23] Ratcliffe E, Gatersleben B, Sowden PT (2020). Predicting the perceived restorative potential of bird sounds through acoustics and aesthetics. Environ. Behav..

[24] Cox DTCC (2017). Doses of neighborhood nature : The benefits for mental health of living with nature. Bioscience.

[25] Jiang B, Li D, Larsen L, Sullivan WC (2016). A dose–response curve describing the relationship between urban tree cover density and self-reported stress recovery. Environ. Behav..

[26] Van den Berg M (2016). Visiting green space is associated with mental health and vitality: A cross-sectional study in four european cities. Heal. Place.

[27] Wood L, Hooper P, Foster S, Bull F (2017). Public green spaces and positive mental health—Investigating the relationship between access, quantity and types of parks and mental wellbeing. Heal. Place.

[28] Dallimer M (2012). Biodiversity and the feel-good factor: Understanding associations between self-reported human well-being and species richness. Bioscience.

[29] Dean J, van Dooren K, Weinstein P (2011). Does biodiversity improve mental health in urban settings?. Med. Hypoth..

[30] Hough RL (2014). Biodiversity and human health: Evidence for causality?. Biodivers. Conserv..

[31] Lovell R, Wheeler BW, Higgins SL, Irvine KN, Depledge MHA (2014). Systematic review of the health and well-being benefits of biodiverse environments. J. Toxicol. Environ. Heal. Part B Crit. Rev..

[32] Aerts R, Honnay O, Van Nieuwenhuyse A (2018). Biodiversity and human health: Mechanisms and evidence of the positive health effects of diversity in nature and green spaces. Br. Med. Bull..

[33] Ruokolainen L (2017). Holistic view on health: Two protective layers of biodiversity. Ann. Zool. Fennici.

[34] Ruokolainen L, Fyhrquist N, Haahtela T (2016). The rich and the poor: Environmental biodiversity protecting from allergy. Curr. Opin. Allergy Clin. Immunol..

[35] Rook GA (2013). Regulation of the immune system by biodiversity from the natural environment: An ecosystem service essential to health. Proc. Natl. Acad. Sci. USA.

[36] Hanski I (2012). Environmental biodiversity, human microbiota, and allergy are interrelated. Proc. Natl. Acad. Sci. USA.

[37] Manes F (2016). Regulating Ecosystem Services of forests in ten Italian Metropolitan Cities: Air quality improvement by PM10 and O3 removal. Ecol. Indic..

[38] Dearborn DC, Kark S (2010). Motivations for conserving urban biodiversity. Conserv. Biol..

[39] Taylor L, Hochuli DF (2015). Creating better cities: How biodiversity and ecosystem functioning enhance urban residents’ wellbeing. Urban Ecosyst..

[40] Li X, Zhou W, Ouyang Z, Xu W, Zheng H (2012). Spatial pattern of greenspace affects land surface temperature: Evidence from the heavily urbanized Beijing metropolitan area, China. Landsc. Ecol..

[41] Wood CL, Lafferty KD (2013). Biodiversity and disease: A synthesis of ecological perspectives on Lyme disease transmission. Trends Ecol. Evol..

[42] Huynen MMTE, Martens P, De Groot RS (2004). Linkages between biodiversity loss and human health: A global indicator analysis. Int. J. Environ. Health Res..

[43] Fuller RA, Irvine KN, Devine-Wright P, Warren PH, Gaston KJ (2007). Psychological benefits of greenspace increase with biodiversity. Biol. Lett..

[44] Carrus G (2015). Go greener, feel better? The positive effects of biodiversity on the well-being of individuals visiting urban and peri-urban green areas. Landsc. Urban Plan..

[45] Marselle MR, Irvine KN, Lorenzo-Arribas A, Warber SL (2016). Does perceived restorativeness mediate the effects of perceived biodiversity and perceived naturalness on emotional well-being following group walks in nature?. J. Environ. Psychol..

[46] Bebbington A (2005). The ability of A-level students to name plants. J. Biol. Educ..

[47] Schebella MF, Weber D, Schultz L, Weinstein P (2020). The nature of reality: Human stress recovery during exposure to biodiverse, multisensory virtual environments. Int. J. Environ. Res. Public Health.

[48] Meng L, Li S, Zhang X (2024). Assessing biodiversity’s impact on stress and affect from urban to conservation areas: A virtual reality study. Ecol. Indic..

[49] Brancato G, Van Hedger K, Berman MG, Van Hedger SC (2022). Simulated nature walks improve psychological well-being along a natural to urban continuum. J. Environ. Psychol..

[50] Robinson MD, Clore GL (2002). Belief and feeling: Evidence for an accessibility model of emotional self-report. Psychol. Bull..

[51] White M (2010). Blue space: The importance of water for preference, affect, and restorativeness ratings of natural and built scenes. J. Environ. Psychol..

[52] Berto R (2005). Exposure to restorative environments helps restore attentional capacity. J. Environ. Psychol..

[53] MacKerron G, Mourato S (2013). Happiness is greater in natural environments. Glob. Environ. Chang..

[54] Shiffman S, Stone AA, Hufford MR (2008). Ecological momentary assessment. Annu. Rev. Clin. Psychol..

[55] Sterne JAC (2009). Multiple imputation for missing data in epidemiological and clinical research: Potential and pitfalls. BMJ.

[56] White IR, Royston P (2009). Imputing missing covariate values for the Cox model. Stat. Med..

[57] Marselle MR (2021). Pathways linking biodiversity to human health: A conceptual framework. Environ. Int..

[58] Luck GW, Davidson P, Boxall D, Smallbone L (2011). Relations between urban bird and plant communities and human well-being and connection to nature. Conserv. Biol..

[59] Shiffman S (2014). Conceptualizing analyses of ecological momentary assessment data. Nicotine Tob. Res..

