# Supplementary material for: Smartphone-based ecological momentary assessment reveals an incremental association between natural diversity and mental wellbeing
Source: Sci Rep. 2024 Apr 16;14:7051. doi: 10.1038/s41598-024-55940-7 (PMC11021539; doi:10.1038/s41598-024-55940-7)
Supplement: Supplementary file 1 — Supplementary Tables. [file 41598_2024_55940_MOESM1_ESM.docx]

**Supplementary Table 1.** Momentary associations between natural features and mental wellbeing using the Multiple Imputation with Chained Equations (MICE) procedure.

|  | **25% response rate (*n* = 1,998)** | | **50% response rate (*n* = 922)** | | **75% response rate (*n* = 310)** | |
| --- | --- | --- | --- | --- | --- | --- |
|  | Unadjusted | Adjusted | Unadjusted | Adjusted | Unadjusted | Adjusted |
|  | **MD**  **(95% CI)** | **MD**  **(95% CI)** | **MD**  **(95% CI)** | **MD**  **(95% CI)** | **MD**  **(95% CI)** | **MD**  **(95% CI)** |
| Seeing or hearing birds | **1.46*****  **(1.33, 1.59)** | **1.45*****  **(1.32, 1.60)** | **1.61*****  **(1.45, 1.78)** | **1.60*****  **(1.43, 1.77)** | **1.55*****  **(1.31, 1.80)** | **1.54*****  **(1.29, 1.78)** |
| Seeing trees | **1.16*****  **(1.04, 1.27)** | **1.14*****  **(1.02, 1.26)** | **1.20*****  **(1.04, 1.36)** | **1.19*****  **(1.03, 1.35)** | **1.15*****  **(0.92, 1.37)** | **1.12*****  **(0.89, 1.35)** |
| Seeing plants | **1.37*****  **(1.25, 1.49)** | **1.36*****  **(1.24, 1.47)** | **1.38*****  **(1.23, 1.53)** | **1.37*****  **(1.22, 1.52)** | **1.34*****  **(1.11, 1.57)** | **1.34*****  **(1.11, 1.58)** |
| Seeing or hearing water | **1.49*****  **(1.31, 1.67)** | **1.47*****  **(1.29, 1.65)** | **1.66*****  **(1.43, 1.88)** | **1.64*****  **(1.42, 1.87)** | **1.60*****  **(1.26, 1.95)** | **1.59*****  **(1.25, 1.93)** |
| *Note: Mean difference (MD) and 95% confidence intervals (CI) represent the mean difference in momentary mental wellbeing per category increase compared to the reference group.*  *Statistically significant associations (p* < 0.05*) are highlighted in bold.*  *All models employed the Multiple Imputation with Chained Equations (MICE) procedure. Analyses were explored as crude associations and after adjusting for age, gender, ethnicity, education, and occupation.*  ** p* < 0.05  *** p* < 0.01  **** p* < 0.001 | | | | | | |

**Supplementary Table 2.** Time-lasting associations between natural features and mental wellbeing.

|  | **25% response rate (*n* = 1,998)** | | **50% response rate (*n* = 922)** | | **75% response rate (*n* = 310)** | |
| --- | --- | --- | --- | --- | --- | --- |
|  | Unadjusted | Adjusted | Unadjusted | Adjusted | Unadjusted | Adjusted |
|  | **MD**  **(95% CI)** | **MD**  **(95% CI)** | **MD**  **(95% CI)** | **MD**  **(95% CI)** | **MD**  **(95% CI)** | **MD**  **(95% CI)** |
| Seeing or hearing birds |  |  |  |  |  |  |
| L0 | **1.54*****  **(1.35, 1.73)** | **1.51*****  **(1.32, 1.70)** | **1.61*****  **(1.39, 1.82)** | **1.58*****  **(1.37, 1.80)** | **1.61*****  **(1.32, 1.90)** | **1.59*****  **(1.30, 1.88)** |
| L1 | **0.58*****  **(0.39, 0.77)** | **0.53*** (0.34, 0.72)** | **0.56*****  **(0.34, 0.77)** | **0.52*****  **(0.31, 0.73)** | **0.54*****  **(0.26, 0.83)** | **0.51*****  **(0.22, 0.80)** |
| L2 | **0.23***  **(0.04, 0.42)** | 0.18  (-0.01, 0.37) | 0.21  (0.00, 0.42) | 0.18  (-0.03, 0.39) | 0.17  (-0.11, 0.46) | 0.14  (-0.14, 0.44) |
| Seeing trees |  |  |  |  |  |  |
| L0 | **1.16*****  **(0.99, 1.34)** | **1.12*****  **(0.95, 1.30)** | **1.09*****  **(0.90, 1.29)** | **1.07*****  **(0.88, 1.27)** | **1.15*****  **(0.88, 1.41)** | **1.12**  **(0.85, 1.39)** |
| L1 | **0.35*****  **(0.18, 0.52)** | **0.30****  **(0.12, 0.47)** | **0.31****  **(0.12, 0.51)** | **0.28****  **(0.09, 0.48)** | **0.41****  **(0.14, 0.68)** | **0.37****  **(0.10, 0.64)** |
| L2 | 0.15  (-0.03, 0.32) | 0.10  (-0.07, 0.27) | 0.00  (-0.19, 0.19) | -0.03  (-0.23, 0.16) | 0.06  (-0.20, 0.33) | 0.03  (-0.24, 0.30) |
| Seeing plants |  |  |  |  |  |  |
| L0 | **1.37*****  **(1.19, 1.55)** | **1.33*****  **(1.15, 1.51)** | **1.31*****  **(1.11, 1.51)** | **1.29*****  **(1.09, 1.50)** | **1.24*****  **(0.97, 1.52)** | **1.23*****  **(0.95, 1.51)** |
| L1 | **0.33*****  **(0.15, 0.51)** | **0.28****  **(0.10, 0.46)** | **0.32****  **(0.12, 0.52)** | **0.29****  **(0.09, 0.49)** | **0.35***  **(0.07, 0.62)** | **0.31***  **(0.03, 0.59)** |
| L2 | -0.05  (-0.23, 0.13) | -0.09  (-0.27, 0.09) | -0.17  (-0.37, 0.03) | -0.20  (-0.40, 0.00) | -0.09  (-0.37, 0.19) | -0.12  (-0.40, 0.16) |
| Seeing or hearing water |  |  |  |  |  |  |
| L0 | **1.59*****  **(1.32, 1.86)** | **1.56*****  **(1.29, 1.83)** | **1.74*****  **(1.44, 2.03)** | **1.73*****  **(1.43, 2.03)** | **1.84*****  **(1.45, 2.23)** | **1.85*****  **(1.45, 2.24)** |
| L1 | 0.16  (-0.11, 0.43) | 0.15  (-0.12, 0.42) | 0.20  (-0.10, 0.49) | 0.19  (-0.11, 0.49) | 0.20  (-0.20, 0.60) | 0.21  (-0.19, 0.61) |
| L2 | 0.18  (-0.08, 0.45) | 0.14  (-0.13, 0.41) | 0.12  (-0.17, 0.42) | 0.09  (-0.21, 0.38) | -0.03  (-0.42, 0.37) | -0.07  (-0.47, 0.32) |
| *Note: Mean difference (MD) and 95% confidence intervals (CI) represent the mean difference in momentary mental wellbeing per category increase compared to the reference group.*  *Statistically significant associations (p* < 0.05*) are highlighted in bold.*  *Analyses were explored as crude associations and after adjusting for age, gender, ethnicity, education, occupation.*  ** p* < 0.05  *** p* < 0.01  **** p* < 0.001 | | | | | | |

**Supplementary Table 3.** Time-lasting associations between natural features and mental wellbeing using the Multiple Imputation with Chained Equations (MICE) procedure.

|  | **25% response rate (*n* = 1,998)** | | **50% response rate (*n* = 922)** | | **75% response rate (*n* = 310)** | |
| --- | --- | --- | --- | --- | --- | --- |
|  | Unadjusted | Adjusted | Unadjusted | Adjusted | Unadjusted | Adjusted |
|  | **MD**  **(95% CI)** | **MD**  **(95% CI)** | **MD**  **(95% CI)** | **MD**  **(95% CI)** | **MD**  **(95% CI)** | **MD**  **(95% CI)** |
| Seeing or hearing birds |  |  |  |  |  |  |
| L0 | **1.59*****  **(1.39, 1.78)** | **1.56*****  **(1.36, 1.75)** | **1.67*****  **(1.46, 1.88)** | **1.65*****  **(1.44, 1.86)** | **1.64*****  **(1.36, 1.93)** | **1.63*****  **(1.35, 1.91)** |
| L1 | **0.56*****  **(0.36, 0.74)** | **0.51*****  **(0.33, 0.70)** | **0.53*****  **(0.33, 0.74)** | **0.50*****  **(0.30, 0.71)** | **0.56*****  **(0.28, 0.84)** | **0.53*****  **(0.25, 0.81)** |
| L2 | **0.20***  **(0.01, 0.39)** | 0.16  (-0.04, 0.35) | 0.17  (-0.04, 0.38) | 0.14  (-0.07, 0.36) | 0.18  (-0.11, 0.46) | 0.15  (-0.13, 0.44) |
| Seeing trees |  |  |  |  |  |  |
| L0 | **1.17*****  **(0.99, 1.34)** | **1.13*****  **(0.95, 1.30)** | **1.10*****  **(0.91, 1.29)** | **1.08*****  **(0.89, 1.28)** | **1.15*****  **(0.89, 1.42)** | **1.12*****  **(0.86, 1.39)** |
| L1 | **0.35*****  **(0.18, 0.52)** | **0.30****  **(0.13, 0.48)** | **0.34****  **(0.15, 0.53)** | **0.31****  **(0.12, 0.50)** | **0.42****  **(0.15, 0.68)** | **0.38****  **(0.11, 0.64)** |
| L2 | 0.14  (-0.04, 0.32) | 0.10  (-0.08, 0.27) | 0.02  (-0.18, 0.22) | -0.01  (-0.21, 0.19) | 0.07  (-0.20, 0.34) | 0.04  (-0.23, 0.31) |
| Seeing plants |  |  |  |  |  |  |
| L0 | **1.38*****  **(1.20, 1.56)** | **1.34*****  **(1.16, 1.52)** | **1.31*****  **(1.11, 1.51)** | **1.30*****  **(1.10, 1.50)** | **1.30*** (1.02, 1.58)** | **1.29*****  **(1.01, 1.56)** |
| L1 | **0.34*****  **(0.17, 0.52)** | **0.30****  **(0.13, 0.48)** | **0.35****  **(0.15, 0.55)** | **0.32****  **(0.12, 0.52)** | **0.35***  **(0.07, 0.62)** | **0.31***  **(0.03, 0.58)** |
| L2 | -0.04  (-0.22, 0.15) | -0.07  (-0.26, 0.11) | -0.13  (-0.33, 0.07) | -0.16  (-0.36, 0.04) | -0.04  (-0.32, 0.23) | -0.08  (-0.36, 0.20) |
| Seeing or hearing water |  |  |  |  |  |  |
| L0 | **1.61*****  **(1.34, 1.88)** | **1.59*****  **(1.32, 1.86)** | **1.75*****  **(1.45, 2.04)** | **1.74*****  **(1.45, 2.03)** | **1.80*****  **(1.41, 2.19)** | **1.80*****  **(1.41, 2.20)** |
| L1 | 0.13  (-0.13, 0.40) | 0.13  (-0.14, 0.39) | 0.11  (-0.18, 0.40) | 0.11  (-0.18, 0.40) | 0.13  (-0.26, 0.54) | 0.15  (-0.25, 0.55) |
| L2 | 0.13  (-0.14, 0.40) | 0.09  (-0.18, 0.36) | 0.06  (-0.24, 0.36) | 0.03  (-0.27, 0.34) | -0.05  (-0.46, 0.35) | -0.10  (-0.50, 0.31) |
| *Note: Mean difference (MD) and 95% confidence intervals (CI) represent the mean difference in momentary mental wellbeing per category increase compared to the reference group.*  *Statistically significant associations (p* < 0.05*) are highlighted in bold.*  *All models employed the Multiple Imputation with Chained Equations (MICE) procedure. Analyses were explored as crude associations and after adjusting for age, gender, ethnicity, education, occupation.*  ** p* < 0.05  *** p* < 0.01  **** p* < 0.001 | | | | | | |

**Supplementary Table** **4.** Associations between natural diversity score, exposure to natural environments, mental wellbeing, and their time-lasting effects using the Multiple Imputation with Chained Equations (MICE) procedure.

|  | **25% response rate (*n* = 1,998)** | | | **50% response rate (*n* = 922)** | | **75% response rate (*n* = 310)** | |
| --- | --- | --- | --- | --- | --- | --- | --- |
|  | Unadjusted | | Adjusted | Unadjusted | Adjusted | Unadjusted | Adjusted |
|  | **MD**  **(95% CI)** | | **MD**  **(95% CI)** | **MD**  **(95% CI)** | **MD**  **(95% CI)** | **MD**  **(95% CI)** | **MD**  **(95% CI)** |
| Total natural diversity score | **0.73*****  **(0.68, 0.78)** | | **0.72*****  **(0.67, 0.77)** | **0.77*****  **(0.70, 0.83)** | **0.76*****  **(0.70, 0.82)** | **0.74*****  **(0.65, 0.83)** | **0.74*****  **(0.64, 0.83)** |
| Time-lasting effects | | |  |  |  |  |  |
| L0 | **0.75*****  **(0.68, 0.82)** | | **0.73*****  **(0.66, 0.81)** | **0.75*****  **(0.67, 0.83)** | **0.74*****  **(0.66, 0.82)** | **0.76*****  **(0.66, 0.87)** | **0.76*****  **(0.65, 0.86)** |
| L1 | **0.20*****  **(0.14, 0.27)** | | **0.18*****  **(0.12, 0.25)** | **0.20*****  **(0.12, 0.28)** | **0.19*****  **(0.11, 0.27)** | **0.23*****  **(0.12, 0.33)** | **0.21*****  **(0.10, 0.32)** |
| L2 | 0.06  (-0.01, 0.14) | | 0.04  (-0.03, 0.12) | 0.03  (-0.05, 0.10) | 0.01  (-0.07, 0.09) | 0.04  (-0.07, 0.15) | 0.02  (-0.08, 0.13) |
| Natural environments | **3.56*****  **(3.29, 3.84)** | | **3.56*****  **(3.28, 3.83)** | **3.89*****  **(3.54, 4.23)** | **3.89*****  **(3.54, 4.23)** | **4.09*****  **(3.59, 4.60)** | **4.10*****  **(3.59, 4.61)** |
| Time-lasting effects | |  |  |  |  |  |  |
| L0 | **3.56*****  **(3.12, 4.01)** | | **3.56*****  **(3.12, 4.01)** | **3.68*****  **(3.20, 4.16)** | **3.69*****  **(3.21, 4.17)** | **4.15*****  **(3.53, 4.78)** | **4.18*****  **(3.55, 4.81)** |
| L1 | **0.98*****  **(0.54, 1.41)** | | **1.00*****  **(0.56, 1.43)** | **1.16*****  **(0.68, 1.63)** | **1.17*****  **(0.69, 1.65)** | **0.96** (0.34, 1.58)** | **0.98****  **(0.36, 1.60)** |
| L2 | 0.36  (-0.07, 0.79) | | 0.38  (-0.06 0.81) | 0.33  (-0.15, 0.81) | 0.33  (-0.15, 0.81) | 0.52  (-0.09, 1.13) | 0.52  (-0.10, 1.14) |
| *Note: Mean difference (MD) and 95% confidence intervals (CI) represent the mean difference in momentary mental wellbeing per category increase compared to the reference group.*  *Statistically significant associations (p* < 0.05*) are highlighted in bold.*  *All models employed the Multiple Imputation with Chained Equations (MICE) procedure. Analyses were explored as crude associations and after adjusting for age, gender, ethnicity, education, and occupation.*  ** p* < 0.05  *** p* < 0.01  **** p* < 0.001 | | | | | | | |
